# Reliability of a Smooth Pursuit Eye-Tracking System (EyeGuide Focus) in Healthy Adolescents and Adults

**DOI:** 10.3390/jfmk8020083

**Published:** 2023-06-16

**Authors:** Alan J. Pearce, Ed Daly, Lisa Ryan, Doug King

**Affiliations:** 1 College of Sport Health Engineering, La Trobe University, Melbourne 3086, Australia; 2 School of Science & Computing, Atlantic Technological University, H91 T8NW Galway, Ireland; ed.daly@atu.ie (E.D.); lisa.ryan@atu.ie (L.R.); 3 Auckland Bioengineering Institute, The University of Auckland, Auckland 1142, New Zealand; doug.king35@gmail.com; 4 Wolfson Research Institute for Health and Wellbeing, Department of Sport and Exercise Sciences, Durham University, Durham DH1 3LE, UK

**Keywords:** oculomotor pursuits, eye-movement, concussion, assessment, learning effect

## Abstract

Mild traumatic brain injury (mTBI) is the most common brain injury, seen in sports, fall, vehicle, or workplace injuries. Concussion is the most common type of mTBI. Assessment of impairments from concussion is evolving, with oculomotor testing suggested as a key component in a multimodality diagnostic protocol. The aim of this study was to evaluate the reliability of one eye-tracking system, the EyeGuide Focus. A group of 75 healthy adolescent and adult participants (adolescents: *n* = 28; female = 11, male = 17, mean age 16.5 ± 1.4 years; adults *n* = 47; female = 22; male = 25, mean age 26.7 ± 7.0 years) completed three repetitions of the EyeGuide Focus within one session. Intraclass correlation coefficient (ICC) analysis showed the EyeGuide Focus had overall good reliability (ICC 0.79, 95%CI: 0.70, 0.86). However, a familiarization effect showing improvements in subsequent trials 2 (9.7%) and 3 (8.1%) was noticeable in both cohorts (*p* < 0.001) with adolescent participants showing greater familiarization effects than adults (21.7% vs. 13.1%). No differences were observed between sexes (*p* = 0.69). Overall, this is the first study to address the concern regarding a lack of published reliability studies for the EyeGuide Focus. Results showed good reliability, suggesting that oculomotor pursuits should be part of a multimodality assessment protocol, but the observation of familiarization effects suggests that smooth-pursuit testing using this device has the potential to provide a biologically-based interpretation of the maturation of the oculomotor system, as well as its relationship to multiple brain regions in both health and injury.

## 1. Introduction

Eye-tracking has been well established to be able to determine cognitive states in neurological health and injury, or with neurological disorders [1,2,3]. Recently, devices that assess oculomotor eye movements have been developed to improve the objectivity and time taken in order to be able to provide a neurological assessment. This in turn has placed oculomotor testing as a convenient technology to assess neurological injuries such as concussion.

Induced by biomechanical force directly or indirectly to the brain [4], a concussion causes an assemblage of transient signs and symptoms representing complex pathophysiological processes across multiple cortical areas of the brain [4]. Moreover, concussion is an evolving injury whereby symptoms can change (or worsen) over time, reflecting the changing physiology as the brain recovers from the insult.

The current consensus on concussion in sports relies heavily on symptom reporting and presentation, prior to further testing such as neurocognitive assessment, and this may be open to manipulation or under-reporting [5,6]. For example, in elite sports, ~6% of male and female professional athletes in the Australian Football League have admitted to hiding symptoms of concussion [7].

Consequently, there is increasing discussion and emerging evidence of the benefits of utilizing supplementary markers within the concussion assessment protocols [8,9,10]. Incorporation of assessments such as eye-movement testing provides objective screening to assist sports club medical staff who may suspect a concussion, or during the recovery phase after a sport-related concussion [11,12].

Eye movements consist of two different components: specifically, saccades and smooth pursuits. Saccades are primarily directed toward stationary targets, such as reading, whereas smooth pursuit is elicited to track moving targets [13]. In humans, while historically saccades and pursuits were thought of as two different mechanisms, with differing neural pathways, more recent findings support the view that the neural processes underpinning saccades and pursuits are intertwined [14]. Consequently, while saccadic eye movements have been studied in concussion, such as the King–Devick test, smooth pursuits and concussion have not yet been fully explored.

Previous animal studies have shown that visual motion information processing relies on several cortical areas identified as the middle temporal area, superior temporal sulcus, and medial superior temporal area [15]. Damage to these areas has shown oculomotor deficits that could have long-term consequences in smooth pursuits [16] and a marked effect on using motion information for accurate initiation of smooth pursuit and saccadic eye movements [17].

While the physiological mechanisms of smooth pursuits are still yet to be fully understood in humans, the cognitive requirements of focus tracking on a moving object are acknowledged [12,18]. Multiple cortical and subcortical areas are involved in eye movement (see review [19]), making eye tracking a suitable proxy for a measurement of cognitive functioning after a concussion [11,20].

One eye-tracking device recently developed is the EyeGuide Focus. The system is portable and simple to utilize involving a 10 s assessment, quantifying oculomotor smooth pursuit or Dynamic Visuomotor Synchronization (DVMS) [12]. DVMS refers to when individuals track a moving visual target and spatial and temporal predictions are utilized to bypass the neural processing delay associated with visuomotor tracking. Predictive timing is important in DVMS, requiring the key component of attentional functioning. Therefore, as concussion can affect attentional functioning, oculomotor smooth pursuits are useful to objectively quantify decrements in attention [21]. An added advantage of smooth-pursuit testing, is that tracking a moving target is not limited by education levels, learning disabilities, socioeconomic status, ethnicity, or other confounding variables that may affect reading ability as per the King–Devick test [22]. There are emerging studies employing the EyeGuide Focus system, not only across concussion management [23,24,25] but also studies in fatigue [26]. However, to date, there are no published studies on the reliability of the system. The aim of this study was to determine intrasession test–retest reliability of an eye-tracking device in the context of smooth pursuit in both adolescent and adult cohorts. A secondary aim was to compare differences in between cohorts.

## 2. Materials and Methods

A sample of 75 healthy participants was recruited (adolescents: *n* = 28; female = 11, male = 17, mean age 16.5 ± 1.4 years; adults *n* = 47; female = 22; male = 25, mean age 26.7 ± 7.0 years). All participants were physically active, but not involved in elite sports. Participants reported no history of concussions or other traumatic brain injury and no pre-existing visual, neurological, or psychiatric conditions, nor were on any medication. The sample size was determined using an a priori power analysis (G*Power V3.1.9.6, Kiel, Germany) [27] for within and between groups of repeated measures with an assumed effect size of *f* = 0.25; α < 0.05; power (1 − β) = 0.95 would require a minimum sample size of 44. Prior to testing, all participants (or their parent) completed voluntary informed consent. Protocols were approved by La Trobe University Human Research Ethics Committee (HEC18207), conforming to the Declaration of Helsinki.

**Figure 1:**
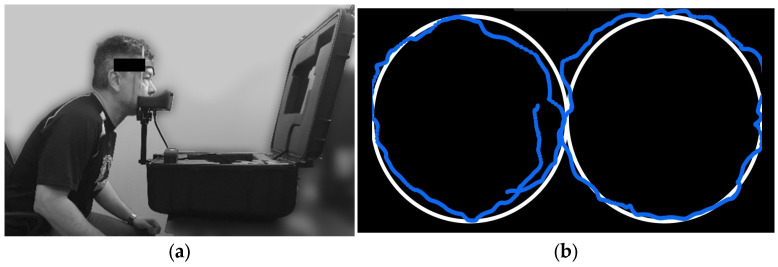
(**a**) Example participant set up for EyeGuide protocol; (**b**) example of participant’s eye-tracking movements (blue) against the stimulus path (white).

Oculomotor smooth-pursuit eye-tracking was completed using the EyeGuide Focus (EyeGuide, Lubbock, TX, USA). The protocol, following EyeGuide protocols, involves the participant sitting with their head placed in a chin/head rest to reduce unnecessary head movements (Figure 1a), and tracking a white dot against a black background moving anticlockwise, then clockwise through one cycle of a horizontal “lazy 8” or “infinity-shape” path for 10 s (Figure 1b). The fixed-position digital camera follows the participant’s pupils, at a 60 Hz frequency, comparing the position and movement speed of the pupils to the white dot stimulus.

Participants undertook testing in a quiet room, away from potential distractions. Participants were also allowed usual eyewear such as prescription contact lenses or glasses, following standard protocol from other eye-movement testing such as the King–Devick [28] as well as recommended protocol from EyeGuide. Each participant completed three trials with a 30 s rest between each trial to avoid potential fatigue. A successful trial was defined as a ‘tick’ indicating that their score fell into the EyeGuide community range [23]. If a trial was unsuccessful, that is, their result was 1SD above the mean, the EyeGuide would indicate the trial was not successful, and participants were allowed to repeat that trial. If three successful trials were not able to be obtained after an arbitrary 10 attempts, the participant’s data was not included. Eye-tracking score was calculated as the cumulative distance between the stimulus data and the pupil position [29]. A greater error was indicated by a larger test score. Participants and test administrators were not blinded to test results, with a classification of scores provided at the end of the test (e.g., “Superior”, “High Average”, “Above Average”, “Average”, “Low Average”, “Impaired”, “Severely Impaired”); however, exact test scores were not displayed. Data collected by the EyeGuide system were imported into Jamovi (V2.3, Sydney, Australia). Data were screened for normal distribution using the Shapiro–Wilk test and found to be normally distributed (W = 0.98, *p* = 0.18). Test–retest reliability was calculated via intraclass correlation coefficient (ICC). ICC values were selected using the criteria by Koo and Li [30]: ≥0.91: excellent reliability, 0.75–0.9: good reliability, 0.51–0.74: moderate reliability, and ≤0.5: poor reliability. Data for ICC are presented as value (±95% CI), and comparisons between groups using mean (±SD). Cohen’s *d* effect sizes were utilized to describe differences between group means [31]. EyeGuide scores are reported in arbitrary units (AUs).

## 3. Results

All but three adult participants completed testing. For three adult participants, data were not able to be collected due to the EyeGuide being unsuccessful in tracking pupil movement.

### 3.1. Test–retest Reliability Analyses

ICCs for all participants (*n* = 72) showed good test–retest reliability across three trials (0.79, 95%CI: 0.70, 0.86). Sub-comparisons between trials 1 and 2, trials 1 and 3, and trials 2 and 3 showed good reliability (ICCs: 0.76 [95%CI: 0.62, 0.84], 0.77 [95%CI: 0.64, 0.85], 0.83 [95%CI: 0.73, 0.89], respectively).

ICCs in all adolescents showed overall good reliability (0.78, 95%CI: 0.62, 0.88) with moderate reliability seen between trails 1 and 2 (0.74, 95%CI: 0.51, 0.87) and between trials 1 and 3 (0.65, 95%CI: 0.35, 0.82). A good reliability was found between trials 2 and 3 (0.81, 95%CI: 0.63, 0.91). When comparing reliability by sex, males showed good overall reliability (0.81, 95%CI: 0.61, 0.82) and across trials 1 and 2, trials 1 and 3, and trials 2 and 3 (0.77 [95%CI: 0.47, 0.91], 0.81 [95%CI: 0.54, 0.92], 0.87 [95%CI: 0.67, 0.95], respectively). In females, overall good reliability was observed (0.76, 95%CI: 0.44, 0.92). A good reliability across trials 1 and 2, and trials 2 and 3 was seen (0.71 [95%CI: 0.22, 0.91], 0.77 [95%CI: 0.35, 0.93], respectively), but poor reliability was seen between trials 1 and 3 (0.48, 95%CI: 0.14, 0.83).

In all adults, ICCs showed overall good test–retest reliability (0.80, 95%CI: 0.67, 0.89) and good reliability between trails 1 and 2 (0.79, 95%CI: 0.61, 0.88), trials 2 and 3 (0.82, 95%CI: 0.67, 0.90), and between trials 1 and 3 (0.84, 95%CI: 0.71, 0.91). When comparing reliability by sex, adult males showed good overall reliability (0.79, 95%CI: 0.60, 0.90) and across trials 1 and 2, trials 2 and 3, and trials 1 and 3 (0.80 [95%CI: 0.58, 0.91], 0.80 [95%CI: 0.50, 0.91], 0.83 [95%CI: 0.63, 0.92], respectively). Similarly, in females, overall good reliability was observed (0.86, 95%CI: 0.73, 0.94) as well as between trials 1 and 2, trials 2 and 3, and trials 1 and 3 (0.86 [95%CI: 0.70, 0.94], 0.86 [95%CI: 0.70, 0.94], 0.84 [95%CI: 0.65, 0.93], respectively).

### 3.2. Comparisons across Trials and Groups

**Figure 2:**
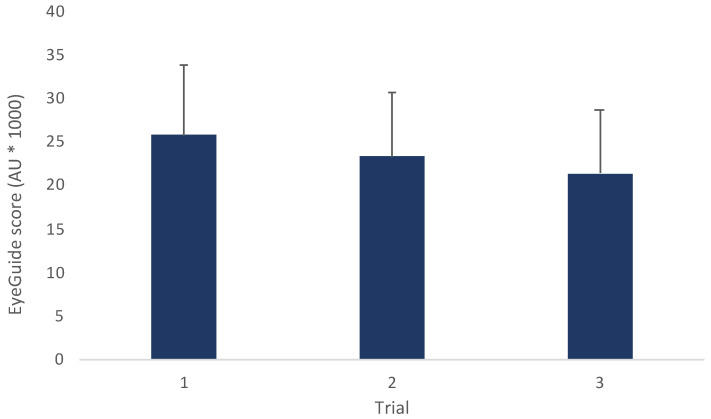
Mean (±SD) for all participants (*n* = 72, mean age 22.7 ± 7.4 years). (*F*_(2,140)_ = 28.2, *p* < 0.001; AU = arbitrary units).

A one-way ANOVA for all participants (*n* = 72) showed a significant improvement in scores with subsequent trials (*F*_(2,140)_ = 28.2, *p* < 0.001; Figure 2). Post hoc comparisons revealed significant differences between trials 1 and 2 (*t*_(71)_ = 4.12, *p* < 0.001, *d* = 0.33), trials 1 and 3 (*t*_(71)_ = 7.21, *p* < 0.001, *d* = 0.57), and trials 2 and 3 (*t*_(71)_ = *t*_71_ = 3.69, *p* = 0.001, *d* = 0.26).

**Figure 3:**
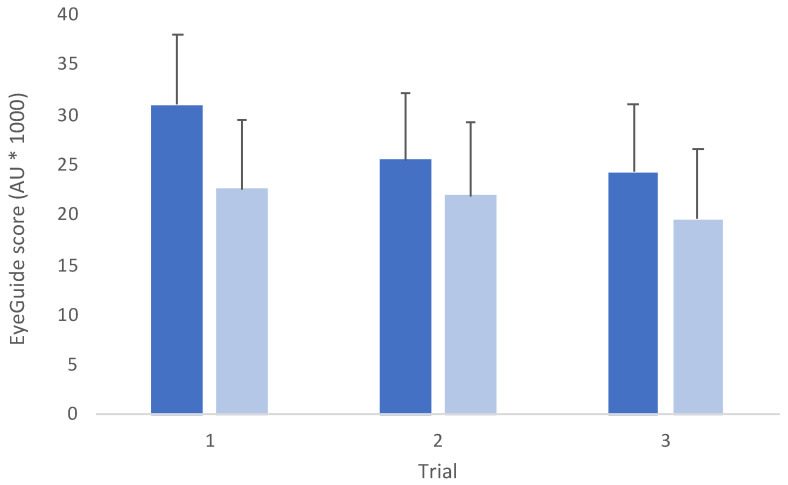
Mean (±SD) across trials for adolescent (blue, *n* = 28) and adult (light blue, *n* = 44) groups. (*F*_(2,140)_ = 10.3, *p* < 0.001; AU = arbitrary units).

Comparison between groups revealed an interaction effect between groups (*F*_(2,140)_ = 10.3, *p* < 0.001; Figure 3). Post hoc testing between groups showed that there was a statistically significant difference for trial 1 (*t*_(70)_ = 4.97, *p* < 0.001, *d* = 1.20), but no differences for trial 2 (*t*_(70)_ = 2.11, *p* = 0.58, *d* = 0.52) or trial 3 (*t*_(70)_ = 2.79, *p* = 0.10, *d* = 0.68).

**Figure 4:**
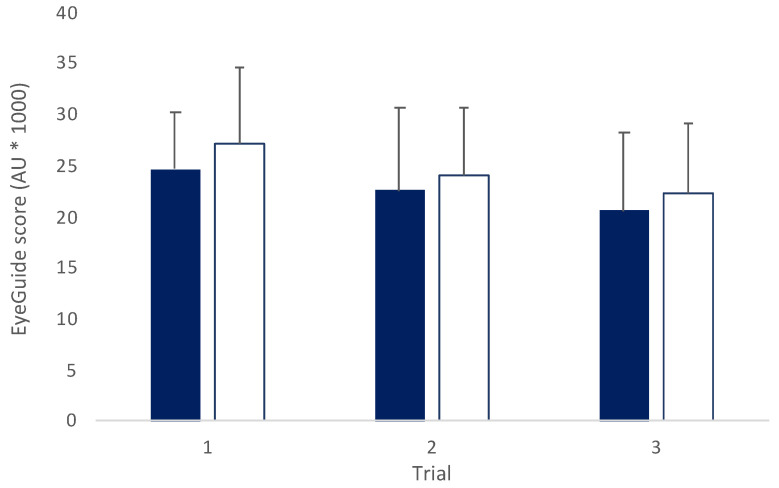
Mean (±SD) across trials for all male (blue solid, *n* = 39) and all female (open, *n* = 33) groups. (*F*_(2,140)_ = 0.36, *p* = 0.69; AU = arbitrary units).

There was no interaction effect found between sexes across trials (*F*_(2,140)_ = 0.36, *p* = 0.69; Figure 4). A main effect was observed for trials (*F*_(2,140)_ = 29.08, *p* < 0.001) with post hoc testing revealing significant difference between trials 1 and 2 (*t*_(70)_ = 4.16, *p* < 0.001, *d* = 0.34) and trials 1 and 3 (*t*_(70)_ = 7.21, *p* < 0.001, *d* = 0.58), and between trials 2 and 3 (*t*_(70)_ = 3.64, *p* = 0.002, *d* = 0.26). No differences were seen between sexes (*F*_(1,70)_ = 1.38, *p* = 0.24).

**Figure 5:**
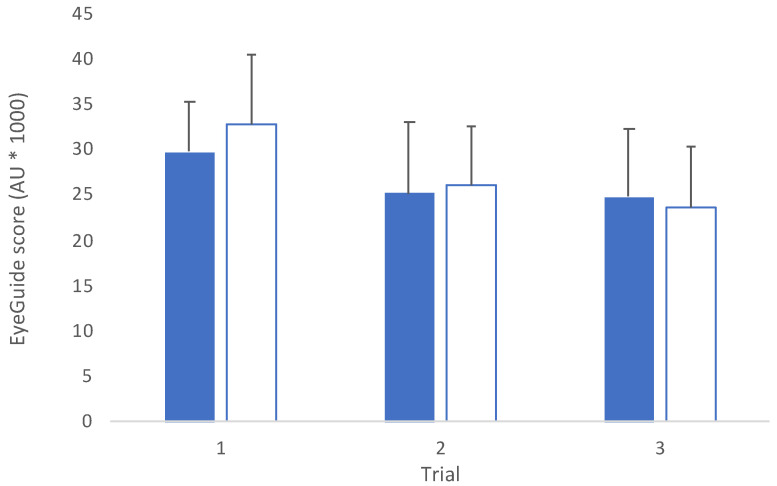
Mean (±SD) across trials for adolescent male (blue solid, *n* = 17) and adolescent female (blue pattern, *n* = 11) groups. (*F*_(2,52)_ = 2.55, *p* = 0.09; AU = arbitrary units).

**Figure 6:**
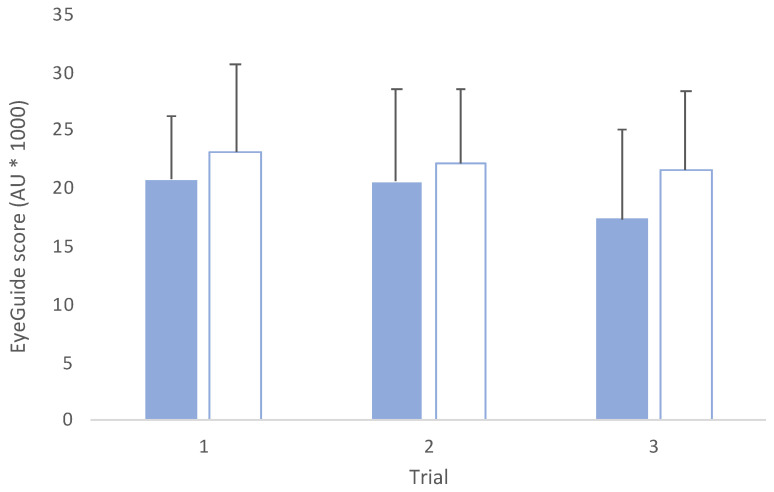
Mean (±SD) across trials for adult male (blue solid, *n* = 22) and adult female (blue pattern, *n* = 22) groups. (*F*_(2,84)_ = 0.99, *p* = 0.37; AU = arbitrary units).

Subanalyses between sexes were conducted in adolescent and adult populations separately (Figure 5 and Figure 6, respectively). In the adolescent group (Figure 5), there was no interaction effect found (*F*_(2,52)_ = 2.55, *p* = 0.09) or main effects between groups (*F*_(1,26)_ = 0.16, *p* = 0.69). A main effect across trials was observed (*F*_(2,52)_ = 30.45, *p* < 0.001) with post hoc comparisons showing significant differences between trials 1 and 2 (*t*_(26)_ = 5.77, *p* < 0.001, *d* = 0.87) and trials 1 and 3 (*t*_(70)_ = 6.55, *p* < 0.001, *d* = 1.04). No difference was seen between trials 2 and 3 (*t*_(70)_ = 1.84, *p* = 0.23, *d* = 0.12).

Similarly, in the adult group (Figure 6), no interaction effect was observed (*F*_(2,84)_ = 0.99, *p* = 0.37) or main effect between groups (*F*_(1,42)_ = 3.14, *p* = 0.08). A main effect across trials was observed (*F*_(2,84)_ = 11.58, *p* < 0.001) with post hoc comparisons showing significant differences between trials 1 and 3 (*t*_(42)_ = 4.78, *p* < 0.001, *d* = 0.36) and trials 2 and 3 (*t*_(42)_ = 3.52, *p*= *0*.03, *d* = 0.27). No difference was seen between trials 1 and 2 (*t*_(42)_ = 0.98, *p* = 0.99, *d* = 0.08).

## 4. Discussion

To the best of our knowledge, this is the first study to examine the intrasession test–retest reliability of an eye-tracking device (EyeGuide Focus) in the context of smooth pursuits. It is important to note that while concussion remains a medical decision, the use of innovative assessments such as oculomotor pursuits will assist the clinician with robust data to improve clinical decision making. Our study quantified reliability and familiarization effects in both adults and adolescents.

Overall findings suggest that the EyeGuide Focus has good reliability, but familiarization effects were observed in both the healthy adolescent and adult cohorts with subsequent attempts. Of note, the familiarization effects in adolescents contributed to the moderate reliability in subsequent trials.

The reliability of the EyeGuide was comparable to other eye movement cognitive tests (e.g., King–Devick). For example, studies in adolescent populations have reported moderate to good ICCs of 0.81 (95%CI: 0.73, 0.87) and 0.91 (95%CI: 0.86, 0.95) [32,33] and in adults, good ICCs of 0.95 (95% CI: 0.91, 0.97) and 0.91 (95%CI: 0.80, 0.96) [28,34]. The data in the present study are also consistent with previously published EyeGuide data. In 2017, Kelly [23] reported a mean score of 29,633.05 (± 9209.83) AUs in adolescents (12–18 years), and this is comparable to our results for trial 1 (30,976.57 ± 7099.87 AUs). In adults, the mean from trial 1 (22,503.33 ± 7014.32 AUs) was equivalent to the ‘high average’ of the EyeGuide normative range (21,066.64 ± 1858.57 AUs) [23]. Of interest, the baseline range in the current study being classified as ‘high average’ was above the baseline reported, ‘average’ classification, in a cohort of surgeons (no raw data presented, however) by Puckett et al. [26]. However, in studies by Kelly [23] and Puckett et al. [26], only a single baseline trial was recorded.

The finding of familiarization/learning effects in this sample is interesting but not surprising. Well described in cognitive psychology, learning/familiarization effects are noteworthy improvements that occur with repetition of a task until scores no longer notably change and achieve stability [35]. The results in this study concur with previous findings in other eye-movement tests (e.g., King–Devick) with improvements in subsequent trials [36]. Consequently, based upon the results of this study, it is suggested that a minimum of three successful trials are completed with the best score used as the player’s baseline.

The data also showed that, overall, adults performed testing with ~20% less error than adolescents (Figure 3). Similar to studies in other eye-movement tests that show improved performance with age [22], these differences reflect the tight relationship between neural maturation and the development of visual functions [37]. For example, adults can accurately pursue moving objects at speeds >30 deg/s [38]. Children’s and adolescents’ accurate tracking of increased smooth-pursuit speeds, while not meeting the same standard as adults, does not improve with age [38,39], suggesting that the mechanisms contributing to differences between adolescents and mature oculomotor systems include longer saccade latencies, reduced smooth-pursuit gain, and increased general variability, reflecting maturation differences between groups [40,41]. Based upon these differences and data from other eye-movement studies that have shown changes with age [22], it is recommended that annual baseline testing be completed in adolescents.

The concern that reliability is affected by improvement in scores, suggesting familiarization effects, should not necessarily be interpreted as a limitation but as useful in developing standardized protocols for future use. The diminishing familiarization effect observed between trials 2 and 3 suggests that three trials should suffice to determine a baseline. Given data are collected within a 10 s time frame, this should not be too onerous on both operator and participant. However, further studies are suggested that could determine the optimal number of baseline trials to reduce familiarization effects, particularly in junior athletes undertaking baseline concussion testing.

Further limitations of this study include the relatively young adult population utilized in this study. We focused on a younger adult sample to emulate the type of adult population likely to be using EyeGuide Focus (athletic cohorts). However, concussion is not a ‘young person injury’ and, indeed, many older athletes participate in contact sports and are at risk of concussion. Future studies should consider assessing the reliability in an older population cohort. We reported three participants failing the assessment. We are unsure why the device was not able to pick up the participant’s pupils; however, this may be something for future studies to address. Nevertheless, with a dropout of only three participants, we feel that this has not compromised the data presented in this study.

While previous studies have investigated the effect of workplace fatigue [26], future studies should also look at the effect of acute exercise and fatigue effects on oculomotor smooth-pursuit tracking. Other eye-movement tests have found an influence of exercise on outcomes [42], and with these systems being suggested as a ‘sideline’ assessment tool to aid medical decisions, the effect of fatigue on eye-movement performance outcomes is not yet known. Other suggestions for future studies would be to quantify intersession reliability. To reflect baseline concussion testing practice, we conducted intrasession testing. However, it is acknowledged that variability, in lieu of suspected concussion or fatigue, would occur across days and further research should report that.

Keeping in mind the current limitations of this study and opportunities for future research, the potential application of the present results suggest that oculomotor testing has a place within the wider neuropsychological assessment for concussion. While this reliability study was focused on smooth pursuits, it is important to note that the oculomotor system reflects inputs from multiple regions of the brain and, consequently, taking into account the importance of learning, the applications of this testing go beyond oculomotor competencies with injury. In other words, using smooth pursuits can provide a more biologically based interpretation of the maturation of the oculomotor system and its relationship with other brain regions, and in health and injury, it can further the methodological advances brough by the present study. In conclusion, this study has demonstrated the reliability of the EyeGuide Focus, suggesting that this system is suitable for concussion recognition. However, with familiarization effects apparent, baseline determination in adults and adolescents should employ a standardized protocol of a minimum of three trials.

## Data Availability

Data are available upon reasonable request from an authorized institution.
